# Late Infective Endocarditis Involving a Gore HELEX® Atrial Septal Occluder Presenting as a Multiple Sclerosis Pseudo-Flare: A Case Report

**DOI:** 10.7759/cureus.112965

**Published:** 2026-07-19

**Authors:** Laura Krause, Nicholas Stancil, Adam Morley, Susan Noe

**Affiliations:** 1 Internal Medicine, Dwight D. Eisenhower Army Medical Center, Augusta, USA; 2 Cardiology, Dwight D. Eisenhower Army Medical Center, Augusta, USA

**Keywords:** atrial septal defects, cardiac device-related infective endocarditis, mssa bacteremia, percutaneous atrial septal defect closure, staphylococcus aureus endocarditis

## Abstract

Atrial septal defects (ASDs) are common congenital heart defects that can be corrected with the placement of minimally invasive occlusion devices. There is a very small risk of perioperative infection associated with these devices following placement. Late endocarditis involving occlusion devices is an exceedingly rare complication of percutaneous occlusion devices. Here we present the case of a female in her 30s who presented with neurologic symptoms typical of her multiple sclerosis flares, but was found to be secondary to late infective endocarditis of an ASD occlusion device requiring surgical excision.

## Introduction

Atrial septal defects (ASDs) are common congenital heart defects that can be successfully treated with percutaneous occlusion devices. This minimally invasive approach has allowed for shorter hospitalizations and quicker recovery times as compared to open surgical correction [1]. The most common complications of percutaneous devices include device embolization, arrhythmia, and thrombosis [2,3]. Infection is less common, typically occurring within three to six months following device placement [3]. Late infective endocarditis (IE) associated with ASD occlusion devices is an exceedingly rare complication occurring more than six months following device placement and is theorized to be related to incomplete endothelialization. There have been only a handful of case reports describing late IE associated with ASD occlusion devices, and less than five reported cases involving Gore HELEX® Septal Occluder devices specifically in the literature to date [2,4].

## Case presentation

A 39-year-old female with a history of relapsing-remitting multiple sclerosis (MS), previously treated with ocrelizumab; methicillin-sensitive *Staphylococcus aureus* (MSSA) osteomyelitis of the left iliac crest following bone graft harvesting for anterior cervical discectomy and fusion (ACDF); and a secundum ASD status post Gore HELEX® occlusion device placement 17 years earlier, presented for evaluation of headache, fever, and neurologic symptoms typical of her prior MS flares, including right upper-extremity weakness and right foot drop, which began on the day of presentation. During evaluation, she was found to be febrile at 39.5°C. Physical examination was notable for a single ulcerative lesion on the right lower lip, clinically concerning for herpes labialis, and an erythematous, pustular, papular rash with small areas of necrotic ulceration on the anterior chest. The patient noted this rash after grooming her dog several days before presentation. Cardiac examination was unremarkable, including no murmurs, rubs, or gallops. Neurologically, strength was diminished to 4/5 on the right upper and lower extremities, but otherwise intact.

Laboratory evaluation and imaging were completed, and blood and urine cultures were drawn. This was notable for leukocytosis of 15.4 × 10^3^/µL with neutrophil predominance, an erythrocyte sedimentation rate of 9 mm/h, and an ultra-sensitive C-reactive protein level of 39.3 mg/dL. The complete metabolic panel, hepatic function panel, creatine kinase, lactic acid level, and urinalysis were unremarkable. Chest X-ray and head computed tomography (CT) were within normal limits. Lumbar puncture was performed in the emergency department. Empiric therapy with ceftaroline, levofloxacin, and IV acyclovir was initiated per infectious disease recommendations.

Cerebrospinal fluid (CSF) analysis was within normal limits, and CSF polymerase chain reaction (PCR) testing was negative for standard meningoencephalitis pathogens, including herpes simplex virus. Both sets of blood cultures, obtained from two separate venipuncture sites, grew MSSA within several hours of incubation. The antimicrobial regimen was narrowed to cefazolin monotherapy. As antibiotic therapy continued, fever abated and neurologic symptoms resolved.

**Figure 1:**
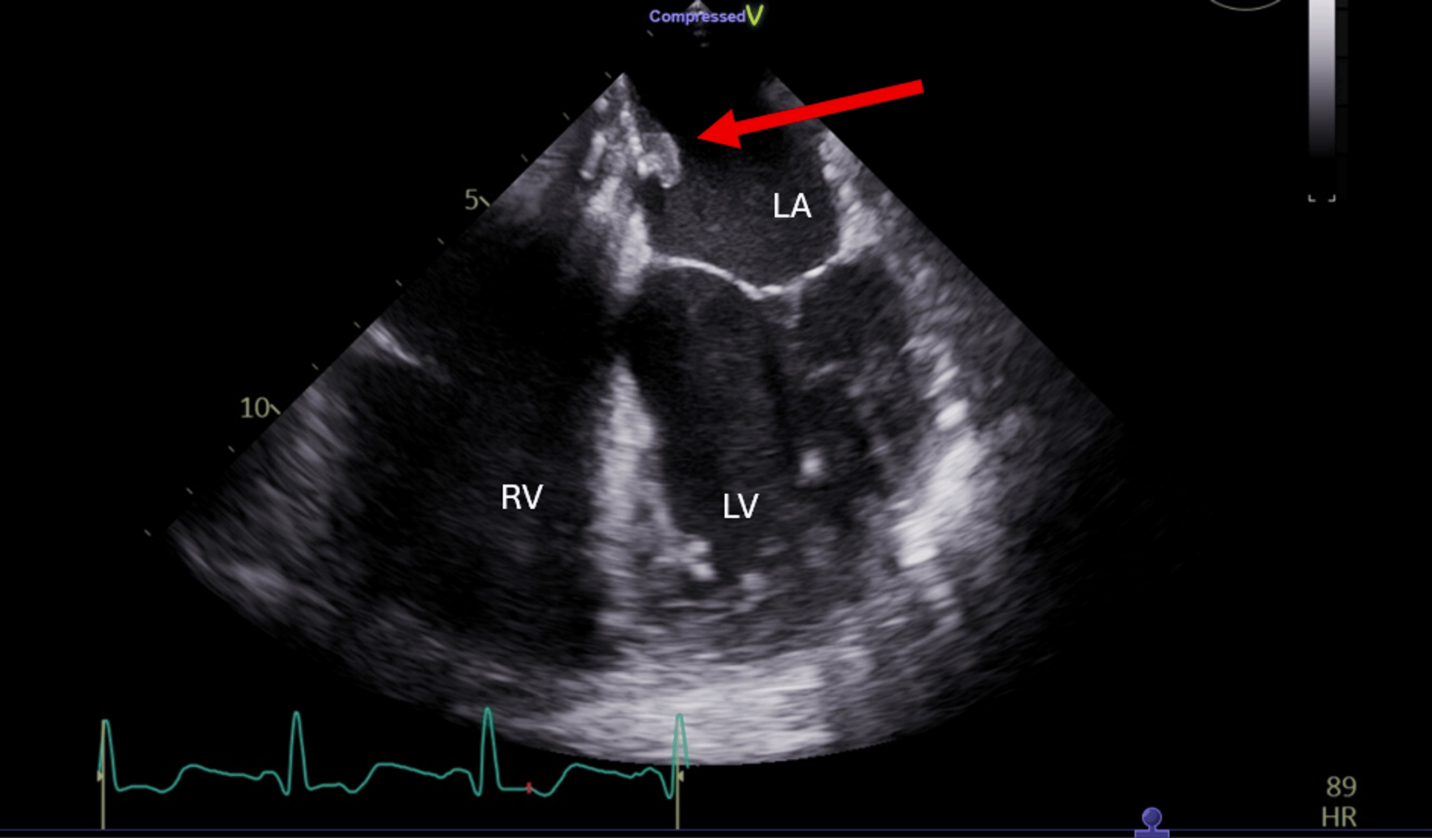
Transesophageal echocardiogram demonstrating a large vegetation adherent to the left atrial side of the atrial septal defect (ASD) occlusion device (red arrow). The device is appropriately positioned within the atrial septum without evidence of device embolization. No valvular pathology, including stenosis, regurgitation, or valvular vegetation, is identified. LA: left atrium; LV: left ventricle; RV: right ventricle

Imaging was obtained as part of the survey for MSSA bacteremia. Transthoracic echocardiogram and subsequent transesophageal echocardiogram demonstrated a large vegetation attached to the left atrial side of the ASD occlusion device without any valvular involvement (Figure 1 and Video 1). Thus, the patient fulfilled both major criteria for definitive diagnosis of IE per the 2000 revised Duke Criteria.

**Video 1:** Transesophageal echocardiogram showing a mobile vegetation on the left atrial side of the atrial septal defect (ASD) occlusion device.

Magnetic resonance imaging (MRI) of the brain with and without contrast demonstrated scattered T2 hyperintensities that were unchanged from prior exams. There was otherwise no evidence of infarct, hemorrhage, mass, or new lesions. MRI of the spine was also obtained. The cervical spine was without evidence of hardware infection or migration. There was no evidence of infection in the native cervical, thoracic, or lumbar spine, including no abscess, discitis, osteomyelitis, or myositis. Left hip MRI was also without evidence of infection, including no evidence of osteomyelitis in the iliac crest.

The patient was transferred to another facility for definitive management of IE. She underwent surgical excision of the occlusion device and two-layer autologous pericardial patch repair of the remnant ASD without complication (Video 2). Vegetation was identified on the left atrial side of the occlusion device, consistent with the vegetation visualized on TEE and clinically consistent with IE. Postoperative TEE demonstrated preserved cardiac function with no leaks in the patch repair. Images from this TEE are unavailable. She was treated with six weeks of cefazolin and three months of warfarin postoperatively. After shared decision-making with the patient, she opted to continue lifelong suppressive therapy with cefadroxil.

**Video 2:** Intraoperative recording showing surgical excision of atrial septal occlusion device and placement of two layer autologous pericardial patch repair of remnant ASD. ASD: atrial septal defect

At the three-month outpatient cardiology follow-up visit, repeat transthoracic echocardiogram demonstrated intact surgical pericardial patch repair with no residual shunting (Video 3). The patient had returned to baseline functional status.

**Video 3:** Three-month postoperative transthoracic echocardiogram. Apical four-chamber view demonstrating an intact pericardial patch repair of the atrial septum. Color Doppler imaging (right panel) showed no evidence of a residual interatrial shunt.

## Discussion

Late IE associated with ASD occlusion devices is an exceedingly rare device complication involving less than 1% of all device placements. This patient developed late IE, 17 years following device placement. Based on currently published case reports, the most remote case of ASD occlusion device-associated late IE occurred 14 years after placement. This case would extend the timeline of known infectious complications of ASD occlusion devices [4].

It is unclear what the source of infection is in this case. She did have a history of MSSA osteomyelitis in the left iliac crest several years prior; however, there were multiple negative blood cultures in the interim, and MRI obtained during evaluation was without evidence of osteomyelitis, so this was thought to be less likely. The patient also had cervical spine hardware following ACDF. Given the prior episode of MSSA bacteremia, this could have been seeded previously and could be a potential source of infection for the current presentation. However, cervical MRI was without evidence of infection, so this was also considered less likely. The active folliculitis noted on her chest had small areas of ulceration, which can be seen when deeper infection of the skin occurs with *S. aureus* folliculitis, and thus could lead to bacteremia [5]. Ultimately, folliculitis was our leading theory as to the source of infection. The patient opted to pursue lifelong suppressive antibiotic therapy due to the lack of confidence in the source of infection, the presence of cervical hardware, and the desire to explore treatment options for MS in the future that would result in immunosuppression.

It is worth discussing the initial consideration of meningoencephalitis. This patient was previously treated with ocrelizumab for MS. The last dose was given approximately one year prior to presentation and was being held due to prior osteomyelitis. Ocrelizumab is typically dosed every six months, so we opted to approach this patient as relatively immunocompromised. Additionally, ocrelizumab carries a post-market warning for being associated with herpes simplex virus encephalitis [6]. The presence of labial ulcerations on presentation that appeared clinically consistent with herpes labialis further raised this concern, but initial CSF analysis was not consistent with infection, and subsequent CSF PCR was negative for herpes simplex virus.

As antibiotic therapy was initiated for MSSA bacteremia, the patient defervesced, and there was a gradual resolution of right-sided weakness. This, in conjunction with stable brain MRI, makes this patient's overall presentation more consistent with MS pseudo-relapse secondary to ASD occlusion device-associated late IE as a consequence of Uhthoff's phenomenon [7].

Definitive management of IE requires surgical excision and debridement, which was completed successfully in this patient. Postoperative warfarin was pursued due to intraoperative atrial fibrillation and as prophylaxis with placement of the pericardial patch. The patient ultimately had an uncomplicated postoperative course and returned to baseline functional status.

## Conclusions

Although the Gore HELEX device has since been removed from the market and advances in bioprosthetic technology continue to improve the efficacy and incorporation of occlusion devices into clinical practice, it is important to remember that patients may still experience complications long after device placement. It is also critical to remember assessment of bioprosthetic devices during evaluation of *S. aureus* bacteremia in addition to traditional “hardware”, as these may also become complicit in infection. This case highlights an atypical presentation of IE as a consequence of a rare surgical complication.
